# Effects of a Yoga Exercise Program on Cognitive Distortion, Communication Skills, Intolerance of Uncertainty, and Psychological Characteristics Among Probationary Employees

**DOI:** 10.3390/bs16081432

**Published:** 2026-08-20

**Authors:** Z. Dilay Ekiz, Elif Karagun

**Affiliations:** 1 Instıtute of Health, Kocaeli University, İzmit 41000, Kocaeli, Türkiye; 2 Sport Science Faculty, Kocaeli University, İzmit 41000, Kocaeli, Türkiye

**Keywords:** psychological characteristics, communication skills, cognitive distortion, intolerance of uncertainty, probation employees

## Abstract

This study aimed to evaluate the effects of a ten-week yoga exercise program, implemented for probation employees, on psychological characteristics, cognitive distortions, communication skills, and intolerance of uncertainty. The Cognitive Distortion Scale, the Brief Symptom Inventory, the Intolerance of Uncertainty Scale, and the Communication Skills Scale were administered to 71 volunteer employees. Thirty-three employees who indicated they would regularly participate in the yoga exercise program were assigned to the intervention group, and 38 employees were assigned to the control group. A quasi-experimental pretest–posttest control group design was employed. Statistical analyses were performed using the SPSS 24 software. The intervention group showed a significant adjusted posttest advantage in communication skills, although the time × group interaction was not significant. Psychological symptom scores showed favorable within-group reductions in anxiety, depression, and hostility in the intervention group; however, the adjusted group and time × group effects for overall symptoms were not significant. The total cognitive distortion scores showed a nonsignificant favorable trend. Conversely, intolerance of uncertainty increased significantly more in the intervention group than in the control group, indicating an unfavorable change. The yoga exercise regimen demonstrated limited and mixed effects in reducing anxiety, depression, and feelings of hostility within a group setting, and improving communication skills. The findings suggest that yoga can be integrated into institutional training and psychological support programs for employees.

## 1. Introduction

Occupations exposed to secondary trauma are characterized by chronic emotional labor, uncertainty, and indirect trauma, which increase vulnerability to psychological strain (Kinman & Grant, 2011; Violanti, 2012). Occupational stress contributes to many diseases and psychosomatic disorders. Yoga practice can effectively neutralize occupational stress (Zok et al., 2024). Probation employees represent a particularly high-risk group due to sustained exposure to high-risk individuals, crisis decision-making, and institutional responsibility. Such conditions are associated with emotional exhaustion, hypervigilance, cognitive overload, and secondary traumatic stress (Çakıroğlu, 2018; Gürdil & Erden, 2016; Kaya, 2015; Kaptanoglu, 1997; Hanlon & Garfinkel, 2010; Muirhead & Fortune, 2016; Ekinci, 2019). Psychological symptoms were conceptualized within the diagnostic framework provided by the DSM-IV-TR (American Psychiatric Association, 2000). Job satisfaction was negatively associated with organizational stress (Başbuğa et al., 2018), while mindfulness-based training was found to reduce work-related stress and improve job satisfaction (Shonin et al., 2014). Probation practice is governed by defined legal duties and professional boundaries (Ataç & Nursal, 2006; Probation Law, 2005; Probation Department, 2005). Probation employees’ occupational context differs from that of general employees and other helping professions, especially with regard to chronic uncertainty, institutional responsibility, crisis-related decision making, and repeated contact with individuals under legal supervision, which may increase emotional exhaustion, hypervigilance, cognitive overload, and secondary traumatic stress (Yavuz, 2012; Kinman & Grant, 2011; Violanti, 2012; Çakıroğlu, 2018; Gürdil & Erden, 2016). 

Therefore, this occupational group requires separate investigation to determine whether yoga may be a feasible supportive intervention for psychological symptoms, cognitive distortions, communication skills, and intolerance of uncertainty in probation settings (Büssing et al., 2012). Although yoga and mindfulness-based interventions have been widely studied in students, clinical groups, and general adult populations, evidence specific to probation employees remains limited (Brown & Gerbarg, 2005; Rocha et al., 2012; Vadiraja et al., 2009; Spinaris et al., 2012; Hillhouse et al., 2023). Stress emerges when occupational demands exceed available psychological and physiological resources, contributing to burnout and maladaptive cognitive processing (Bakker & Demerouti, 2007). Recreational activities are recommended for coping with negative emotions such as trauma, correcting cognitive distortions, improving mood, and establishing healthy relationships. It has been suggested that these recommended activities, especially when practiced in groups, support social interaction (Oner Suata & Karagün, 2022; Karagün et al., 2010; Koç, 2007). Individuals need to develop different strategies to manage the challenges they face (Baltaş & Baltaş, 1990). The formation and maintenance of maladaptive coping strategies, core beliefs, conditioned assumptions, automatic thoughts, and personality patterns all influence our cognitive framework (Beck et al., 2004). In this context, mind–body interventions have gained attention as regulatory approaches targeting both physiological and psychological stress systems. Yoga practice has been associated with autonomic nervous system regulation, increased parasympathetic activation, vagal modulation, and reductions in anxiety and depressive symptoms (Brown & Gerbarg, 2005; Streeter et al., 2012; Field, 2011). These effects are more appropriately conceptualized as calming and regulating our body’s general stress system (hormones and nervous system) of the neuroendocrine stress response rather than specific neurotransmitter claims. Beyond emotional symptoms, yoga and mindfulness-based practices have been linked to cognitive flexibility and reduced maladaptive belief patterns (Gard et al., 2014; Baer, 2003). Cognitive distortions and intolerance of uncertainty are established predictors of anxiety and stress vulnerability in high-demand occupational settings (Briere, 2000; Carleton, 2016; Dugas et al., 2001). From a neurocognitive perspective, an improved regulatory balance may reduce threat appraisal and enhance perceived control in uncertain environments. Emerging evidence also suggests that enhanced vagal regulation supports interpersonal functioning and social engagement (Porges, 2011; Luberto et al., 2018).

Yoga and mindfulness practices are often conducted with students, clinical cases, the general adult population, and on workers who exhibit high rates of secondary trauma (Spinaris et al., 2012). By evaluating this intervention in a high-risk occupational group prone to burnout and secondary trauma, such as probation officers, this study addresses a critical gap in the occupational mental health literature (H. P. Smith et al., 2022). Most research typically focuses on baseline measures such as stress, heart rate, and overall well-being. Unique stressors include heavy workloads, uncertainty, health and safety risks, role ambiguity, sleep-disrupting shift schedules, lack of autonomy, lack of variety, inmate/client assault and manipulation, and conflicts with coworkers (Hillhouse et al., 2023). Our study examined the direct impact of yoga on deep mental and cognitive schemas, such as intolerance of uncertainty and cognitive distortion, establishing a link between somatic (bodily) practice and cognitive psychology. Some research suggests that effective mental health and well-being are comparable to other commonly used interventions, such as cognitive behavior therapy (CBT) or antidepressants (Goyal et al., 2014; Kuyken et al., 2015; Dike et al., 2021). Evidence also suggests that the benefits of manualized mindfulness programs extend to memory and some aspects of executive functioning, cognitive flexibility, and meta-awareness (Lao et al., 2016). Yoga may reduce anxiety, depression, and hostility, which may indirectly support communication. Rather than proposing a reductive conclusion regarding the universal efficacy of the intervention, this study offers nuanced and empirical findings. This provides a more transparent and realistic contribution to the literature by demonstrating the non-linear effects of psychological interventions.

The present study may contribute to the literature by evaluating yoga as a structured self-regulation intervention for professionals operating in demanding environments to achieve cognitive flexibility, emotional regulation, and impulse control (specifically regarding rage). This study provides a theoretical framework for how yoga’s “present moment” practice physiologically extinguishes the “threat perception” (amygdala response) in the face of uncertainty (Violanti, 2012; Kinman & Grant, 2011; Bakker & Demerouti, 2007). Yoga may be particularly suitable for this population; as it targets autonomic regulation, vagal tone, and stress-related somatic tension (Streeter et al., 2012; Brown & Gerbarg, 2005; Porges, 2011). Compared to conventional aerobic or strength-based exercises, yoga additionally enhances interoceptive awareness and emotion regulation capacities (Farb et al., 2015; Mehling et al., 2012; Ross & Thomas, 2010). Emerging evidence suggests that yoga and mindfulness-based practices may support interpersonal functioning. Regular practice has been associated with improved communication skills, empathy, and prosocial tendencies (Luberto et al., 2018; Donald et al., 2019). Mindfulness-related cognitive flexibility may reduce helplessness and intolerance of uncertainty by decreasing threat perception and strengthening perceived control (Wong et al., 2023; Carleton, 2016). Although physiological measurements were not performed in the current study, the literature suggests that vagal regulation and autonomic balance may be behind these changes. Neurophysiological models further propose that yoga modulates autonomic balance activity, contributing to emotional regulation and stress resilience (Streeter et al., 2012; Pascoe & Bauer, 2015). Building on biopsychosocial and embodied regulation frameworks, this study conceptualizes yoga as an intervention operating across physiological, cognitive, and relational domains. It evaluated whether a 10-week yoga program produces differences in psychological symptoms, cognitive distortions, communication skills, and intolerance of uncertainty among probation employees working in high-stress institutional settings. Our model examines whether regular yoga practice produces significant differences between probation employees who participate in yoga and those who do not, in high-stress occupational settings. Specifically, it investigates whether yoga facilitates transformations across interconnected domains.

Based on the multi-component framework of the study, hypotheses regarding systemic changes in interconnected domains—level of psychological/mental symptoms, cognitive and emotional difficulty toward ambiguous regulation, cognitive distortions, and communication processes—were formulated as follows: consistent yoga practice will not result in notable variations in psychological regulation, cognitive distortions, or communication processes between probationers who engaged in the intervention and those who did not, nor will it demonstrate enhanced psychological regulation, diminished cognitive distortion, or favorable communication processes. In other words, can a structured physical intervention generate systematic changes in cognitive and interpersonal functioning? From an applied perspective, yoga may be integrated into institutional training programs, and psychological support services could be restructured within a multicomponent framework combining yoga and psychoeducation.

## 2. Materials and Methods

### 2.1. Study Population

The study population consisted of public institution employees (psychologists, sociologists, social workers, teachers, preachers, and administrative staff). This context was chosen because probation officers work in a high-stress institutional environment characterized by professional responsibility, demands related to uncertainty, and regular interactions with individuals under legal supervision. The study was conducted during the pandemic, a period in which health-related uncertainty, changes in workload, and social restrictions may have further increased occupational stress (Brooks et al., 2020; Skaalvik & Skaalvik, 2010).

Prior to participant recruitment, ethical approval was obtained from the relevant institutional ethics committee (Approval No. 2021/130), and institutional permission was obtained from the Ministry of Justice. Following these approvals, preliminary information meetings were held at the institution to provide information about the purpose of the study, procedures, voluntary participation, and confidentiality principles. The informed consent form was explained, sent in writing to everyone, and written informed consent was obtained from the employees who agreed to participate.

Eligibility criteria were defined before participant recruitment. The study included volunteers who were active full-time employees at the probation institution, attended the in-house information meeting, and provided written informed consent. Participants were informed that they could withdraw from the study at any time, without penalty. Invitations to the project information meeting were declared to approximately 250 personnel working in the two provincial directorates. (n = 33 yoga participants completed both pre- and posttests; n = 38 control participants completed both pre- and posttests.)

Employees who were unable to participate in mild-to-moderate physical exercise due to chronic medical or physical conditions, who were on extended leave for the duration of the 10-week study, were excluded.

After meeting dates were determined at the relevant institutions where the study would be conducted, the consent form was read aloud. Consent forms were left for each employee who came to the information area to read the forms. Consent forms were carefully collected to ensure the confidentiality of volunteer participants. The forms signed by the volunteer participants were collected.

Allocation was non-random and based on voluntary participation. A quasi-experimental pretest–posttest control group design was used. Group allocation was non-random and based on willingness to participate in the yoga program, and assignment was based on voluntary participation in the yoga program. In addition, the application of quasi-experimental designs in non-artificial, natural environments (e.g., corporate structures, classrooms) is advantageous because it provides researchers with a practical framework for establishing cause-and-effect relationships (Creswell & Creswell, 2018). Furthermore, quasi-experimental designs used for causal inference have high external validity because they are conducted in natural environments outside the laboratory, such as workplaces, providing generalizability to real-world environments (Shadish et al., 2002).

Employees who agreed to participate in the 10-week yoga exercise program were assigned to the intervention group, while those who did not participate were assigned to the control group. The final sample consisted of 71 employees: 33 in the intervention group and 38 in the control group. Because the assignment was non-random and was based on voluntary participation, the findings should be interpreted with potential self-selection bias and underlying group differences. The sensitivity of pandemic conditions was considered in our study because they increased stress levels due to uncertainty, health risks, workload changes, and social isolation (Brooks et al., 2020).

### 2.2. Setting and Recruitment Procedure

The study was conducted in relevant public institutions, providing a familiar and accessible environment for the participants. Participation was initiated through institutional announcements, information brochures, and introductory seminars detailing the scope, multi-component framework, and the voluntary nature of the yoga program. Yoga mats and cleaning supplies were provided by the researcher. An a priori power analysis was not conducted prior to data collection; instead, the sample size was determined by the total number of eligible and willing volunteers available at the institution during the study period. A 10-week yoga intervention was conducted in person by a certified instructor for employees of a probation institution. To ensure validity and pandemic safety, sessions were held twice a week in groups of five, scheduled according to participants’ workloads. Each session had a standard structure including a 15 min dynamic warm-up, breath control, a basic sequence of traditional asanas, and 15 min of mindfulness and relaxation. The program, announced to approximately 250 people at the provincial directorate, saw 71 volunteers participating. There were no withdrawals. Participation was closely monitored, and make-up sessions were coordinated; consequently, 71 participants completed the study.

The implementation flow was as follows:

After compiling information for the project, reviewing the literature, and holding meetings, the project application form was prepared and submitted to the university ethics committee with a faculty approval request letter → ethics approval → institutional permission → information meeting → eligibility assessment → written informed consent → enrollment and workload scheduling → baseline/pretest assessment → non-random group allocation → 10-week yoga intervention/control condition → Week 1: Theoretical and practical intro → Weeks 1–10: Bi-weekly yoga sessions (with make-up tracking) → implementation of yoga practices → posttest assessment → data analysis.

**Participation flow**

Employees informed about the study (approximately n = 250) ↓

Provided written consent and completed baseline assessment (n = 71) ↓

Yoga group (n = 33)

Control group (n = 38)

Session Structure

15 min dynamic warm-up and stretching → core practice (structural alignment, breath and asanas) → 15 min systematic mindfulness and relaxation.

**10-Week Intervention Asana Sequence Flow**

Tadasana (Mountain) pelvic alignment, core engagement → Urdhva Hastasana upper extremity extension → Uttanasana hip flexion, axial elongation → Ardha Uttanasana thoracic extension → transitional plank lowering tailbone, deep abdominal stabilization → Chaturanga Dandasana isometric quadriceps and core, forearms parallel → Urdhva Mukha Svanasana (Upward Dog) scapular retraction, lifting lower abdomen → Adho Mukha Svanasana controlled breaths shoulder stabilization, posterior chain elongation → reverse sequence (Ardha Uttanasana → Uttanasana → Urdhva Hastasana → Tadasana).

**Intervention:** The sessions were attended in groups of five. During the first week of the intervention, the voluntary participants received a concise theoretical and practical introduction covering fundamental yoga principles, alignment techniques, body control, asanas (postures), and pranayama (breathing exercises). Each subsequent session followed a standardized multi-component structure, beginning with 15 min of dynamic stretching and warm-up exercises. This was followed by core practice, which focused on structural alignment, breath control, and the execution of traditional asanas. Each session concluded with a 15 min systematic mindfulness and relaxation practice. Over the 10-week intervention period, the mastery of foundational yoga mechanics, somatic control, and breath awareness was progressively emphasized. Following the completion of the 10th week, all 33 participants in the intervention group and all 38 participants in the control group completed the posttest assessments. No participants withdrew, no posttest questionnaires were missing, and all 71 participants were included in the analyses. The data collection phase has been completed, and posttests have been administered to all participants in the intervention and control groups. Attendance was monitored during the intervention. To ensure ecological validity and minimize occupational disruption, participants enrolled voluntarily and initiated the program within the same calendar week, with sessions scheduled around their professional workloads. The manualized yoga intervention was administered twice weekly for 10 weeks by a certified instructor who monitored attendance and coordinated make-up sessions for missed lessons necessitated by workload fluctuations or pandemic-related adjustments. The core asana sequence was applied repetitively in each session and comprised the following standardized structural components: Mountain Pose (Tadasana), where participants established pelvic alignment and core engagement; Upward Salute Pose (Urdhva Hastasana), emphasizing upper extremity extension and cervical relaxation; Uttanasana, involving hip flexion and axial elongation toward the floor; Half Standing Forward Bend (Ardha Uttanasana), targeting thoracic extension; Tree Pose (Vrikshasana), adapted into a transitional plank alignment to lower the tailbone and engage the deep abdominal stabilizers; Four-Limb Staff Pose (Chaturanga Dandasana), requiring isometric quadriceps and core activation with forearms parallel; Upward-Facing Dog (Urdhva Mukha Svanasana), focusing on scapular retraction and lower abdominal lifting; and Downward-Facing Dog (Adho Mukha Svanasana), maintained for five controlled breaths to facilitate shoulder girdle stabilization and posterior chain elongation. The sequence was systematically concluded by retracing the positions in reverse order (Ardha Uttanasana, Uttanasana, Urdhva Hastasana, and Tadasana) to ensure neurological and physiological grounding.

### 2.3. Research Hypothesis

This study hypothesizes that a 10-week yoga intervention comprising physical postures, breathing techniques, and meditation produces significant improvements in physiological regulation, cognitive distortions, and social communication skills among probation employees, compared to a control group that did not practice yoga.

### 2.4. Assessments

A quantitative methodology was developed using noninvasive clinical practices at the Kocaeli University Health Sciences Institute. An information survey consisting of 165 questions was prepared, based on a literature review, to measure cognitive distortions, communication skills, intolerance of uncertainty, and various sociodemographic factors that affect psychological characteristics.

The Cognitive Distortion Scale, the Intolerance of Uncertainty Scale, the Communication Skills Scale, and the Brief Symptom Inventory were administered to 71 employees. Of these, 33 employees who indicated that they would regularly participate in the yoga exercise program were assigned to the intervention group, while 38 employees were assigned to the control group. The researcher, who was also a yoga instructor, implemented the yoga exercise program for 33 individuals in the intervention group, adhering to pandemic circumstances.

#### 2.4.1. Cognitive Distortion Scale

The Cognitive Distortion Scale was developed by Briere to assess maladaptive cognitive schemas related to self-perception. It has a 40-question, five-point Likert-type scale (e.g., 1 = strongly disagree, 5 = strongly agree). The scale consists of five subscales: Self-Evaluation (negative and inadequate self-perception), Self-Blame, Helplessness, Hopelessness, and seeing life (the future) as dangerous (Ağır & Yavuzer, 2018). Regarding its psychometric properties in the Turkish sample, construct validity was confirmed through factor analysis. Furthermore, the scale suggested strong internal consistency, with a total Cronbach’s alpha coefficient of 0.90 and subscale alphas ranging from 0.75 to 0.88 (Ağır & Yavuzer, 2018). Higher scores indicate high maladaptive cognitive schemas. This means that the person blames themselves more, feels more intense helplessness and hopelessness, and perceives life as more dangerous (representing a negative situation).

#### 2.4.2. Intolerance of Uncertainty Scale

The Intolerance of Uncertainty Scale was developed by Dugas, Freeston, Letarte, Rhéaume, and Ladouceur to measure individuals’ cognitive, emotional, and behavioral responses to uncertain situations; the Intolerance of Uncertainty Scale is a five-point Likert-type scale consisting of 26 questions. The scale consists of four subscales: uncertainty is upsetting and stressful, uncertainty is unfair, uncertainty prevents action, and uncertain events are negative and should be avoided (Sarı & Dağ, 2009). In the Turkish adaptation study, construct validity was supported by a confirmatory factor analysis. The scale showed excellent reliability, with a total Cronbach’s alpha internal consistency coefficient of 0.93 and a high test–retest reliability coefficient of 0.86 (Sarı & Dağ, 2009). Higher scores indicate that the individual experiences high levels of discomfort and stress in the face of uncertainty. It indicates that the individual exhibits negative/anxious cognitive, emotional, and behavioral responses to uncertain situations (i.e., negative situations).

#### 2.4.3. Communication Skills Scale

The Communication Skills Scale was developed by Korkut-Owen and Bugay to assess individuals’ perceived communication skills. This scale employs a five-point Likert-type format and comprises 25 questions. The scale consists of four subscales: communication principles and basic skills, self-expression, willingness to communicate, active listening, and nonverbal communication (Korkut-Owen & Bugay, 2014). The validity and reliability of the Turkish version were established by Korkut-Owen and Bugay (2014). Construct validity analyses indicated a good model fit for the subscales, and the instrument has robust internal consistency, yielding a total Cronbach’s alpha coefficient of 0.88 (Korkut-Owen & Bugay, 2014). Higher scores indicate a high score on this scale reflects a positive situation. This shows that the individual perceives their own communication skills (active listening, self-expression, nonverbal communication, etc.) as developed and adequate.

#### 2.4.4. Brief Symptom Inventory

The Brief Symptom Inventory was developed in 1994 by Leonard R. Derogatis, and is a shortened form of the longer SCL-90-R (Symptom Checklist-90-Revised) scale (Derogatis, 1994). The BSI is a screening instrument used to assess psychological symptom severity and screen psychological well-being and symptom burden in academic research with high-stress occupational groups (It is a comprehensive tool designed to assess individuals’ emotional states. While the original English version measures nine symptom dimensions, the validated Turkish adaptation developed by Sahin and Durak (1994) structures the 53 items into five specific subscales: anxiety, depression, negative self-concept (referred to in some local contexts as negative self-image), somatization, and hostility. This five-factor structure was strictly utilized and scored in this study, in accordance with the psychometric properties established for the Turkish population. Respondents rated items on a 5-point Likert-type scale ranging from 0 (not at all) to 4 (extremely). To capture overall psychological distress, the Global Severity Index (GSI) was calculated using the BSI Total score. The GSI was computed by summing the scores of all 53 items and dividing the total by 53, representing the overall depth and frequency of psychiatric symptoms. Higher GSI scores indicate greater psychological distress. The Turkish version has demonstrated high validity and reliability in clinical and non-clinical samples. In the study’s reference sample, internal consistency (Cronbach’s alpha) coefficients for the five subscales ranged between 0.71 and 0.85, while the overall instrument associated an excellent GSI alpha coefficient of 0.96 (Sahin & Durak, 1994). Higher scores indicate that the frequency and severity of the psychological symptoms (anxiety, depression, somatization, hostility, etc.) experienced by the individual are high. We obtained ethics approval (No. 2021/130) and institutional permissions. Employees were informed under pandemic conditions as stress levels increased due to uncertainty, health risks, workload changes, and social isolation (Brooks et al., 2020), and they provided written consent. No participants withdrew, no posttest questionnaires were missing, and all 71 participants were included in the analyses. The design was a quasi-experimental pretest–posttest control-group design. Our study has the Kocaeli University Faculty of Medicine non-interventional research board approval code. Generative artificial intelligence (GenAI) has been used in this paper.

### 2.5. Data Analysis

The data were analyzed in line with the research objectives. Descriptive statistics were calculated for the sociodemographic variables. Continuous variables were summarized using means (M), standard deviations (SD), and 95% confidence intervals (CI), whereas categorical variables were summarized using frequencies (n) and percentages (%) (Can, 2020) Normality assumptions were assessed prior to the inferential analyses. For within-group comparisons, paired-samples *t*-tests were used for normally distributed data and Wilcoxon signed-rank tests for non-normal data. For between-group comparisons, independent-samples *t*-tests, Mann–Whitney U tests were conducted. Time × group effects were examined using two-way mixed analysis of variance/linear mixed-effects analysis/change-score analysis. The significance level was set at *p* < 0.05. Given the quasi-experimental design with voluntary group allocation, potential self-selection bias, baseline differences, and gender imbalance should be considered when interpreting the results. Accordingly, the findings are presented as preliminary rather than definitive causal evidence.

## 3. Findings

Data were obtained from the intervention and control groups before and after a 10-week yoga program that Provincial Directorate of Probation employees attended in accordance with the research design. Data were compared and analyzed for the intervention group that participated in the yoga program and the control group. The mean scores from the Cognitive Distortion Scale, the Intolerance of Uncertainty Scale, the Brief Symptom Inventory, and the Communication Skills Scale were analyzed to evaluate the effects of the exercise program on cognitive distortions, intolerance of uncertainty, psychological symptoms, and communication skills. Participants voluntarily enrolled in the program and began in the same week without interfering with their work schedules. The intervention consisted of 33 participants, and the control group of 38 participants. Group allocation was based on voluntary participation; no matching procedure was implemented. A significant gender difference was observed between groups (58% female in the intervention group vs. 15% in the control group). Other sociodemographic variables (age, marital status, education level, income, and prior yoga experience) did not differ significantly (*p* > 0.05). Continuous variables were analyzed using independent samples *t*-tests (or Mann–Whitney U tests, where appropriate), and categorical variables were analyzed using chi-square tests.

Table 1 presents the sociodemographic information of the volunteers participating in the study. The chi-square test analysis of the sociodemographic characteristics of the intervention and control groups indicated no significant differences regarding age, marital status, parental status, monthly income, educational attainment, years of employment, and prior yoga experience. Both groups had similar characteristics. Statistical analyses of the Brief Symptom Inventory demonstrated significant findings (*p* < 0.05). The total score showed result, supported by significant pre-test and post-test measurements (*p* < 0.001). Significant variance was also recorded in several sub-dimensions, namely Anxiety at pre-test (*p* = 0.034), Depression at pre-test (*p* = 0.011), and overall Hostility (*p* = 0.027). The gender distribution is explained in the limitations section.

As shown in Table 2, the total Cognitive Distortion Scale score was treated as the primary outcome. The mean total score decreased from M = 1.90, SD = 0.47 at pretest to M = 1.66, SD = 0.48 at posttest in the intervention group, whereas the control-group mean remained relatively stable, changing from M = 1.78, SD = 0.30 to M = 1.79, SD = 0.43. The within-group changes were not statistically significant in either the intervention group, *p* = 0.058, or the control group, *p* = 0.598. After adjustment for baseline scores, the group effect was also not significant; F (1, 68) = 1.11, *p* = 0.296, ηp^2^ = 0.016. The time-by-group change estimate was ΔM = −0.218, 95% CI [−0.498, 0.062], with Hedges’ g = −0.36 and ηp^2^ = 0.034. Because the confidence interval included zero, the changes did not differ significantly between the groups. Overall, the intervention was not associated with a statistically significant reduction in total cognitive distortion scores. For Negative Self, the within-group change was not significant in the intervention group, *p* = 0.381, but was significant in the control group, *p* = 0.006. Nevertheless, the baseline-adjusted group effect was not significant, F (1, 68) = 0.28, *p* = 0.599, ηp^2^ = 0.004, and the time-by-group confidence interval included zero; ΔM = −0.193, 95% CI [−0.543, 0.157]. Thus, the significant within-group change observed in the control group did not constitute evidence of a differential intervention effect. Similarly, for Self-Blame, the intervention group did not show a significant within-group change, *p* = 0.391, whereas the control group showed a significant change, *p* = 0.006. However, the adjusted group effect was not significant; F(1, 68) = 1.48, *p* = 0.227, ηp^2^ = 0.021. The time-by-group estimate was also not significant; ΔM = −0.179, 95% CI [−0.515, 0.156], Hedges’ g = −0.25. For Helplessness, the intervention-group mean increased from M = 1.65, SD = 0.44 to M = 1.87, SD = 0.69, and this within-group change was statistically significant, *p* = 0.046. The corresponding change in the control group was not significant, *p* = 0.607. Because higher Cognitive Distortion Scale scores indicate more maladaptive cognitive schemas, the increase in Helplessness represents an unfavorable rather than a beneficial change. The table reports a change estimate of ΔM = −0.435, 95% CI [−0.775, −0.095], Hedges’ g = −0.60, and ηp^2^ = 0.086. No statistically significant within-group differences were observed for Hopelessness or Seeing the Future as Dangerous. For the latter subscale, the adjusted group effect was not significant, F(1, 68) = 0.19, *p* = 0.666, ηp^2^ = 0.003, and the time-by-group estimate was close to zero, ΔM = 0.017, 95% CI [−0.393, 0.427], Hedges’ g = 0.02. Analysis of the Cognitive Distortion Scale revealed statistically significant results based on a 0.05 significance level. Specifically, differences were observed in the scale’s total score (*p* < 0.001), as well as in the Negative Self/Self-Blame (*p* = 0.006) and Helplessness (*p* = 0.046) sub-dimensions. Overall, the only notable finding was the unfavorable increase in Helplessness in the intervention group. 

The Brief Symptom Inventory total score was evaluated as the primary psychological-symptom outcome. The intervention-group mean decreased from M = 0.76, SD = 0.48 at pretest to M = 0.62, SD = 0.35 at posttest, whereas the control-group mean increased slightly from M = 0.47, SD = 0.39 to M = 0.51, SD = 0.41. Although the table reports statistically significant within-group changes in both groups, *p* = 0.027 and *p* = 0.018, respectively, the baseline-adjusted group effect was not significant; F(1, 68) = 0.45, *p* = 0.503, ηp^2^ = 0.007. The time-by-group estimate was ΔM = −0.129, 95% CI [−0.373, 0.115], with Hedges’ g = −0.25 and ηp^2^ = 0.016. The confidence interval included zero, and the total symptom change did not differ significantly between the groups. For Anxiety, the table reports an adjusted group effect of F(1, 68) = 3.18, *p* = 0.079, ηp^2^ = 0.045. The time-by-group effect was also not significant; F(1, 69) = 0.00, *p* = 0.964, ΔM = −0.006, 95% CI [−0.260, 0.249], Hedges’ g = −0.01. Therefore, anxiety scores did not change differently across the two groups. For Depression, the intervention group showed a significant within-group reduction, *p* = 0.048, whereas the control group did not; *p* = 0.806. However, the adjusted group effect was not statistically significant, F(1, 68) = 0.45, *p* = 0.503, ηp^2^ = 0.007, and the time-by-group confidence interval included zero; ΔM = −0.220, 95% CI [−0.536, 0.096]. For Negative Self, neither the within-group comparisons nor the adjusted group effect was significant, F(1, 68) = 0.53, *p* = 0.471, ηp^2^ = 0.008. For Somatization, the intervention group showed a significant within-group reduction, *p* = 0.016, whereas the control group did not; *p* = 0.821. For Hostility, the adjusted group effect was not significant; F(1, 68) = 0.47, *p* = 0.495, ηp^2^ = 0.007. The time-by-group estimate was also nonsignificant; ΔM = −0.171, 95% CI [−0.475, 0.132], Hedges’ g = −0.27, ηp^2^ = 0.018. Overall, some favorable within-group reductions were observed in the intervention group, but these findings were not supported by statistically significant adjusted group or time-by-group effects.

The total Communication Skills Scale score was analyzed as the primary communication outcome. The intervention-group mean increased slightly from M = 4.16, SD = 0.36 at pretest to M = 4.22, SD = 0.72 at posttest, whereas the control-group mean decreased from M = 3.91, SD = 0.94 to M = 3.86, SD = 0.96. The within-group changes were not statistically significant in either the intervention group, *p* = 0.161, or the control group, *p* = 0.694. After adjustment for baseline scores, however, the group effect was statistically significant, F(1, 68) = 4.83, *p* = 0.031, ηp^2^ = 0.066, indicating a moderate adjusted posttest difference between the groups. In contrast, the time-by-group estimate was small and nonsignificant; ΔM = 0.102, 95% CI [−0.503, 0.708], Hedges’ g = 0.08, ηp^2^ = 0.002. Thus, the adjusted posttest scores favored the intervention group, but the magnitude of pretest-to-posttest change did not differ significantly between the groups. For Communication Principles and Basic Skills, the adjusted group effect was significant, F(1, 68) = 4.57, *p* = 0.036, ηp^2^ = 0.063. However, the time-by-group confidence interval included zero, ΔM = 0.084, 95% CI [−0.536, 0.705], Hedges’ g = 0.06, suggesting that the result reflected an adjusted posttest difference rather than a differential change over time. For Self-Expression, the adjusted group effect was not significant; F(1, 68) = 0.12, *p* = 0.729, ηp^2^ = 0.002. The time-by-group effect was also negligible; ΔM = −0.074, 95% CI [−0.694, 0.546], Hedges’ g = −0.06, ηp^2^ = 0.001. Significant adjusted group effects were observed for Willingness to Communicate and Active Listening, F(1, 68) = 7.99, *p* = 0.006, ηp^2^ = 0.105, and for Non-Verbal Communication; F(1, 68) = 6.08, *p* = 0.016, ηp^2^ = 0.082. Nevertheless, the confidence intervals for the time-by-group estimates included zero in both cases. These findings therefore indicate differences in adjusted posttest levels rather than statistically significant differences in pretest-to-posttest change.

The total Intolerance of Uncertainty Scale score was treated as the primary outcome. The intervention-group mean increased from M = 2.31, SD = 0.83 at pretest to M = 3.11, SD = 0.84 at posttest, whereas the control-group mean increased only slightly from M = 2.16, SD = 0.91 to M = 2.26, SD = 0.90. After adjustment for baseline scores, the group effect was statistically significant and large; F(1, 68) = 16.37, *p* < 0.001, ηp^2^ = 0.194. The time-by-group estimate was also substantial; ΔM = 0.724, 95% CI [0.235, 1.213], Hedges’ g = 0.69, ηp^2^ = 0.112. Because higher scores indicate greater intolerance of uncertainty, this finding represents an unfavorable increase in the intervention group rather than an improvement. For Factor 1, “Uncertainty Is Stressful and Upsetting,” the adjusted group effect was significant; F(1, 68) = 12.35, *p* < 0.001, ηp^2^ = 0.154. The time-by-group estimate was ΔM = 0.554, 95% CI [0.021, 1.088], Hedges’ g = 0.49, ηp^2^ = 0.059. For Factor 2, “Negative Self-Evaluations Related to Uncertainty,” the adjusted group effect was also significant; F(1, 68) = 13.23, *p* < 0.001, ηp^2^ = 0.163, with ΔM = 0.712, 95% CI [0.209, 1.215], Hedges’ g = 0.66, and ηp^2^ = 0.104. For Factor 3, “Not Knowing the Future Is Unsettling,” the adjusted group effect was significant; F(1, 68) = 14.48, *p* < 0.001, ηp^2^ = 0.176. The time-by-group estimate was ΔM = 0.761, 95% CI [0.269, 1.254], Hedges’ g = 0.73, ηp^2^ = 0.121. The largest effect was observed for Factor 4, “Uncertainty Prevents Me from Taking Action,” F(1, 68) = 20.39, *p* < 0.001, ηp^2^ = 0.231, with ΔM = 0.985, 95% CI [0.397, 1.573], Hedges’ g = 0.79, and ηp^2^ = 0.139. Statistical analyses of the Brief Symptom Inventory demonstrated significant findings (*p* < 0.05). The total score showed result, supported by significant pre-test and post-test measurements (*p* < 0.001). Significant variance was also recorded in several sub-dimensions, namely Anxiety at pre-test (*p* = 0.034), Depression at pre-test (*p* = 0.011), and overall Hostility (*p* = 0.027). In our study, total scores differed between groups at both time points (*p* < 0.05). This finding indicates an unfavorable increase in the intervention group. A comprehensive theoretical framework is offered for the possibility of different baseline scores due to groups coming from natural environments in quasi-experimental designs (Shadish et al., 2002). This can be explained by the immediate effect of situational differences (Spielberger et al., 1983). It also highlights the relationship between communication anxiety and individual background (McCroskey, 1977).

Overall, the yoga intervention produced limited and mixed effects. No significant baseline-adjusted group effect was found for total cognitive distortion or overall psychological symptom scores. Although some favorable within-group reductions were observed in psychological symptom subscales, these were not confirmed by significant between-group or time × group effects. An unfavorable increase in Helplessness was also observed. Communication skills showed a significant baseline-adjusted posttest advantage for the intervention group; F(1, 68) = 4.83, *p* = 0.031, ηp^2^ = 0.066. However, the time × group effect was not significant, indicating that the magnitude of change did not differ between groups. In contrast, intolerance of uncertainty increased significantly more in the intervention group; F(1, 68) = 16.37, *p* < 0.001, ηp^2^ = 0.194, ΔM = 0.724, 95% CI [0.235, 1.213]. Because higher scores indicate poorer outcomes, this finding represents an unfavorable intervention-related change. However, because higher scores indicate greater intolerance of uncertainty and therefore less favorable outcomes, this finding should not be interpreted as improvement. Instead, it indicates a greater increase in intolerance of uncertainty in the intervention group relative to the control group. These findings suggest that the yoga program was associated with limited and mixed effects across psychological and interpersonal domains. Given the quasi-experimental design, voluntary group allocation, and baseline differences between groups, the results should be interpreted as preliminary rather than definitive evidence of intervention efficacy.

## 4. Discussion and Conclusions

This study examined the effects of a 10-week yoga intervention on psychological symptoms, cognitive distortions, communication skills, and intolerance of uncertainty among probation employees.

The results for the Cognitive Distortion Scale, presented in Table 2, indicate that the subscales assessing Negative Self-Esteem, Self-Blame, Helplessness, Hopelessness, and Seeing the Future as Dangerous showed statistically significant results when comparing the intervention and control groups in both the pretest and posttest on the Cognitive Distortions subscales. There was a notable decrease in the control group from pretest to posttest in Negative Self-Esteem and Self-Blame scores. The decrease in total cognitive distortion scores is consistent with the idea that yoga/mindfulness components may provide nonsignificant numerical benefits to cognitive processing. However, the lack of significant change in most subdimensions suggests that the cognitive content (schematic interpretation patterns) domain may be more “resistant” to short-term interventions. The unexpected increase in the Helplessness subdimensions necessitates two alternative explanations: (i) in the context of the pandemic, face-to-face practice may have increased helplessness assessments by enhancing the perception of an “uncontrollable threat”; (ii) it may reflect a process challenging, difficult, distressing increased self-monitoring, greater awareness of stress responses, pain sensations, and early emotional/cognitive cuesdescribed in the mindfulness/contemplation literature (Baer, 2003; Lindahl et al., 2017). In contrast, empirical evidence suggests helplessness can be reduced with increased cognitive flexibility and perceived control (Wong et al., 2023). Therefore, the increase observed in this study should not be interpreted as a “permanent negative effect” without long-term follow-up and replication in non-pandemic conditions. Research indicates the therapeutic impact of yoga exercise regimens on cognitive distortions across many professional groups, especially among educators and academics (Ruotsalainen et al., 2015). It has been noted as being very effective in alleviating depression among educators. Instructors and academic personnel who have a reduced susceptibility to pressure also recognize an increased self-efficacy in managing difficult academic circumstances (Pajares, 2002; Skaalvik & Skaalvik, 2010). Studies have indicated statistically significant associations among the subscales of Self-Evaluation, Self-blame, Helplessness, Hopelessness, and Seeing the future as dangerous among educators (Duras, 2022). The improvement (in our study, which could not be directly measured) in the control group may have stemmed from the “Hawthorne Effect” or self-monitoring processes exhibited by participants due to their involvement in the research process. While the intervention group experienced emotional confrontation through yoga, the control group’s stability or improvement in some parameters may be related to defense mechanisms provided by continuing routine functioning during the period of social isolation. Other possible environmental factors include the repeated measurement effect, acclimatization to the test, in-house support, and the time effect. McCarney et al. (2007) and Adair (1984) explain the positive changes (Social Desirability) that individuals in the control group unconsciously exhibit during the process of “comparison with the experimental group” or “self-evaluation”. Lancee et al. (2008), in their study examining the wait-list control group self-evaluation effect, found that even the expectation of improvement among participants was a contributing factor at the beginning. This shows that it can alleviate symptoms. Reduced cognitive distortions result from enhanced mental health, self-efficacy, and coping skills in response to life experiences; these factors jointly influence cognitive styles and the handling of events and stressful academic situations (Bulut et al., 2020). Regular exercise increases serotonin levels and positively affects mood (Baer et al., 2005). The increase in helplessness may reflect heightened self-awareness developed during yoga practice (Yıldırım, 2025). Mind–body interventions can initially increase perceived vulnerability as participants confront suppressed stress. Mindfulness-based interventions may initially cause individuals to become “sensitized” to repressed negative emotions, which can raise scores in the short term. Lindahl et al. (2017) describe in their comprehensive study “The varieties of contemplative experience,” detailing the unexpected increase in anxiety, helplessness, and the surfacing of traumatic memories (challenging phenomena) experienced by participants during meditation and yoga practices. Kabat-Zinn (1990), argues that the contact a person makes with their own pain at the beginning of the mindfulness process may initially be perceived as a “burden”.

Analysis of the Brief Symptom Inventory subscale scores presented in Table 3 revealed no significant findings for the Negative Self-Esteem and Somatization subscales, either in within-group comparisons of intervention and control groups from pretests to posttests or between-group comparisons of the two groups at pretests and posttests. When the total scores on the Brief Symptom Inventory were compared between the pretests and posttests of the intervention and control groups, significant differences were found within each group. The anxiety, depression, and hostility subscale scores on the Brief Symptom Inventory between the intervention and control groups showed a significant difference before the intervention. Within-group reductions were observed, but between-group intervention effects were not significant. Research on relevant studies reveals that combining cognitive behavioral therapy with Kundalini yoga has yielded positive results for anxiety, depression, and sleep quality in individuals with generalized anxiety disorder (Khalsa et al., 2015). Yoga may positively influence cognitive functions, particularly in stress regulation and neurocognitive efficiency (Voss et al., 2022). Similar results were observed in an acute intervention that compared a single 30 min session of meditative (Hatha) yoga with a 30 min session of power (Vinyasa) yoga (Marshall et al., 2020). The meditative yoga session significantly reduced anxiety scores (Marshall et al., 2020; Varkal, 2019; Voss et al., 2022). Research indicates that those who engage in advanced yoga practice have markedly enhanced mindfulness and spiritual well-being and a reduction in mental symptoms, including depression (Gaiswinkler & Unterrainer, 2016). Previous research findings on the benefits of a yoga program exist, but other studies suggest that the advantages of participating in weekly yoga sessions, when done only once a week, may be restricted, and that external factors could impact the outcomes (Montes et al., 2025). During times of crisis, such as a pandemic, internal solidarity (social support) may have enabled the control group personnel to conserve their resources. Yoga is not just a physical exercise; it is a holistic approach that directly activates the parasympathetic nervous system through breath control (pranayama) and meditation. It is a neuro-physiological intervention that balances the nervous system. By stimulating the vagus nerve, it activates the parasympathetic nervous system and provides “emotion regulation” in high-stress occupational groups such as probation officers. Studies indicate that a single 1 h session of physical activity leads to the release of serotonin and results in positive outcomes for reducing muscle strain and physical discomfort typically experienced in the workplace, ultimately having a beneficial impact on somatic issues (Woodyard, 2011). The level of stress in a person’s professional life is substantial, and the notable difference in depression and burnout rates between individuals who engage in physical activity at work and those who do not highlights the importance of such activity (Sane et al., 2012). A strong negative correlation was observed between psychological symptom levels and psychological resilience levels among healthcare staff in a private hospital (Cevizci & Müezzin, 2019). As psychological symptoms decrease, psychological resilience increases; conversely, as psychological symptoms worsen, psychological resilience decreases. Optimism, psychological well-being, workplace conditions, communication style, event perception, perceived social support, familial relationships, and personality traits affect psychological resilience, with beneficial levels of these factors shown to impact the length of an individual’s resilience (Karacaoğlu & Köktaş, 2016; Zhang et al., 2020). During the COVID-19 outbreak, employees with direct exposure exhibited elevated levels of anxiety and sadness compared to those without direct contact. Burnout, anxiety, depression, work satisfaction, and other characteristics have been documented as correlating with heightened sensations of burnout (Arpacıoğlu et al., 2021).

Upon examining the score analyses of the Communication Skills Scale listed in Table 4, there were insignificant differences between the pretests and posttests in both the intervention and control groups. Reviewing the overall mean scores on the Communication Skills Scale indicates that the intervention group showed favorable communication skills after the yoga exercise program, whereas the control group had a decrease in communication skills in the posttest average. Substantial results in communication skills were not observed in the subtests, and there was no significant difference between the assessments before and after the intervention, indicating that reduced communication is associated by the pandemic’s social distancing measures, combined with the participants’ face-to-face yoga practice during this period, may have had an advantage on the intervention group. It would be prudent to consider the planning of comprehensive studies to achieve more conclusive outcomes. This approach can significantly enhance communication skills, job satisfaction, and stress management for employees across various professions (Duyan, 2020). The pattern for communication skills—improvement at the total score level without robust subscale changes—may reflect that interpersonal functioning improves downstream of emotional regulation and self-awareness but can require longer exposure or complementary interpersonal skills training to produce subscale-level change. Meta-analytic evidence indicates that mindfulness-based interventions may improve empathy, although the magnitude of change may vary according to intervention characteristics and empathy dimensions (Hu et al., 2022), which is conceptually linked to communication quality. Nevertheless, pandemic social distancing, reduced workplace interaction opportunities, and reliance on self-report measures may have limited observable interpersonal subscale changes in the present study. It is sensible to conduct longitudinal studies that focus on occupational and long-term impacts, as well as cultural models. Researchers noticed a correlation between stress and communication skills in healthcare professionals, observing fluctuations in pulse rates when comparing pre- and post-physical activity measurements, in addition to a notable rise in self-compassion subscales (Nishimura et al., 2021).

Examination of the Intolerance of Uncertainty Scale scores presented in Table 5 showed insignificant differences between the pretest and posttest values in both the intervention and control groups. Nevertheless, a comparison of the scores between the two groups revealed considerable disparities in both the pretest and posttest results. Insignificant differences were found in the subscales of “Factor 2: Negative self-evaluations about uncertainty” and “Factor 3: Disturbance from uncertainty about the future” upon analysis of the intolerance of uncertainty scale subscales. Analysis of the Communication Skills Scale total scores revealed statistically significant difference between the pretest and post-test measurements (*p* < 0.001). Literature reviews demonstrate that intolerance to uncertainty, when combined with low mindfulness, increases symptoms of depression, and that mindfulness practices can help cope with intolerance to uncertainty (Altan-Atalay et al., 2025). Intolerance to uncertainty is posited as a detrimental predictor of psychological resilience, diminishing individuals’ psychological fortitude (Duru et al., 2022; Karataş & Tagay, 2021; Satici et al., 2021). Research indicate that facing unresolved problems and their rising occurrence reduces people’s tolerance, resulting in greater impatience with uncertainty and ultimately leading to functional impairments (Joshi & Galvin, 2022). Research has shown that psychological resilience can increase the ability to tolerate uncertainty (Lee, 2019).

The results show that yoga has an advantage in stress management. It is generally stated that participants seek to reduce stress through yoga practice (Pascoe et al., 2017; Phansikar et al., 2023; van der Kolk et al., 2014). Participants who have practiced yoga for years are less likely to report a need for stress reduction (Zok et al., 2024). Balakrishnan et al. (2023) attribute this to a better developed parasympathetic nervous system in yogis. The parasympathetic dominance shown in the yoga group suggests that hatha yoga practitioners may be at lower risk for stress-related comorbidities (Balakrishnan et al., 2023). Akdeniz and Kastan (2023) concludes his research by suggesting that yoga should be introduced at a young age and practiced regularly, as it offers a simple solution to significant problems such as pain, anxiety, sleep, and stress that negatively affect our daily lives (Zok et al., 2024). In an investigation of psychological resilience during the pandemic, Vinkers et al. (2020), suggested that maintaining or re-establishing daily routines and developing adaptive coping strategies may support psychological resilience and help mitigate the negative mental health effects of prolonged stress and uncertainty. Elliott et al. (2021), emphasize that the changes seen in control groups during the pandemic are related to the easing of restrictions in the outside world or the stabilization of social support (even digital). Regarding intolerance of uncertainty, the findings did not support a beneficial effect of the intervention. Instead, intolerance of uncertainty increased more in the intervention group than in the control group. Intolerance of uncertainty is a transdiagnostic vulnerability factor closely linked to emotion regulation processes (Sahib et al., 2023), and evidence-based treatments can meaningfully reduce intolerance of uncertainty (Miller & McGuire, 2023). A recent doctoral study conducted with in Türkiye demonstrated that mindfulness skills have been described as a mechanism linking anxiety and intolerance of uncertainty (Parlar Yazıcı, 2024). Taken together, yoga may not directly “remove” uncertainty but may strengthen self-regulation resources that prevent escalation of uncertainty-related distress during high-uncertainty periods. Studies have demonstrated that yoga and mindfulness practices can improve the mental health and interpersonal skills of healthcare workers, with individuals experiencing notable decreases in anxiety levels and undesirable emotions (La Torre et al., 2020). Professionals who participate in physical activity demonstrate enhanced empathy towards their participants, improved comprehension of their requirements, and more effective interactions. Calmness is identified as the most critical change variable in alleviating symptoms (Cayoun & Shires, 2020). A six-week study of university employees discovered that yoga and tai chi resulted in reduced levels of anxiety, depression, and stress, as well as enhanced life satisfaction and self-confidence (Valdesalici et al., 2024). Analysis of the Intolerance of Uncertainty Scale revealed statistically significant results based on a 0.05 significance level. Significant differences were observed in the scale’s total scores for both the pre-test (*p* = 0.026) and post-test (*p* = 0.001) measurements. Furthermore, significant differences were found in specific sub-dimensions, namely Factor 1 (Uncertainty is stressful and upsetting; *p* = 0.039) and Factor 4 (Uncertainty prevents me from taking action; *p* = 0.033. 

Overall, the findings suggest favorable within-group reductions in anxiety, depression, and hostility, a nonsignificant favorable trend in total cognitive distortions, and a significant adjusted posttest advantage in communication skills. These findings are interpreted within an integrated psychophysiological regulation pathway (autonomic regulation → emotional stabilization → cognitive processing → interpersonal functioning) and occupational stress. Probation employees are likely to experience high emotional stress, uncertainty, and chronic situations. Such sustained demands can deplete resources and increase symptom burden, whereas interventions that strengthen personal self-regulation resources may support recovery and functioning (Bakker & Demerouti, 2007).

According to Bennetts (2022), yoga can contribute to reducing psychological distress and increasing psychological well-being by supporting transdiagnostic processes such as mindfulness, self-compassion, attention, and emotion regulation; however, the proposed mechanisms need to be confirmed by experimental studies. Stress-related impairment is driven by resource loss cycles, and resource-building practices may interrupt these cycles (Hobfoll, 1989). Cramer et al. (2013) demonstrated the effectiveness of yoga primarily in individuals with pain and functional impairment.The observed reductions in anxiety, depression, and hostility are broadly consistent with evidence that workplace yoga and yoga-based interventions.

Limitations include the quasi-experimental design with self-selection, modest sample size, single-site delivery, and pandemic-specific stressors. Future research should prioritize randomized trials with active controls, longer follow-up, and mixed-methods components to clarify mechanisms and explain unexpected outcomes such as increases in helplessness. The study’s quasi-experimental nature has led to criticisms regarding cause-and-effect relationships and potential bias. While the quasi-experimental study offers advantages, the fact that group assignments were voluntary inevitably led to self-selection bias. This may have created a basis for motivational and baseline psychological differences and expectancy effects between the intervention and control groups. In particular, the inequality in gender distribution between the groups necessitates an interpretation of the results as a preliminary associative observation. Consecutive *t*-tests were conducted on the same sample for four different scales and dozens of sub-dimensions belonging to them. This increases the risk of finding significance (Type I Error/False Positive). It is recommended that future research utilize randomized assignment and initially match participants’ sociodemographic characteristics. The absence of an a priori sample size calculation and the modest convenience sample may have resulted in available samples, and may have limited the insufficient power ability to detect between-group and interaction effects.

A significant limitation of this study is the asymmetry in gender distribution between the intervention and control groups. While comparative analysis showed that this demographic difference did not reach absolute statistical significance (*p* = 0.057), a significant descriptive imbalance was present: the intervention group had a higher proportion of female participants (n = 19), while the control group was predominantly male (n = 6). This suggests potential structural selection and confounding variance. The high concentration of female participants in the intervention group may have inadvertently influenced the treatment and potentially enhanced the observed improvements in psychological well-being, cognitive schemas, and communication skills. This controlled study, conducted in high-stress institutional settings such as law enforcement and justice personnel, clearly demonstrated that the effect of mindfulness/somatic interventions on stress and psychological outcomes did not vary by gender (no gender moderation effect) (Christopher et al., 2018). Predominance of women in the included samples limited the generalizability of the findings to men (Cramer et al., 2013). They reported that gender variance did not affect/impair the main clinical and psychological recovery outcomes in intervention programs conducted in personnel working under high-strain environments. Stratified sampling by gender or controlling for gender as a key covariate could be considered to define intervention mechanisms more clearly (B. W. Smith et al., 2011; Des Jarlais et al., 2004; Hoffmann et al., 2014). It is still difficult to completely separate the pure therapeutic benefits of 10 weeks of yoga practice from gender-specific stress-recovery models. Stratified sampling by gender or controlling for gender as a key covariate could be considered to allow for a clearer definition of intervention mechanisms.

The findings largely support the study hypotheses, suggesting that yoga operates as a multilevel self-regulation intervention affecting physiological, emotional, cognitive, and interpersonal domains. The negative ideas and emotional states induced by expert cautioning about social distancing and contact avoidance to mitigate disease transmission during the pandemic were considered limitations of the study.

## Figures and Tables

**Table 1:** Sociodemographic characteristics in the intervention and control groups.

Sociodemographic Variables of Participants		Intervention Group		Control Group		*p*	X^2^ (df, N)
		N	%	N	%		
Gender	Woman	19	58%	6	15%	0.057	χ^2^ (1, N = 68) = 13.52
	Man	14	42%	32	85%		
Marital Status	Married	30	91%	35	92%	0.59	χ^2^ (1, N = 68) = 0.03
	Single	3	9%	3	8%		
Monthly Income	5000–7000	7	21%	11	29%	0.42	χ^2^ (4, N = 68) = 4.99
	7001–9000	4	12%	3	8%		
	9001–11,000	10	30%	18	47%		
	11,001–13,000	8	24%	4	11%		
	13,001 and above	4	12%	2	5%		χ^2^ (7, N = 68) = 4.67
Age	18–22	1	3%	0	0%	0.096	
	23–27	1	3%	0	0%		
	28–32	6	18%	5	13%		
	33–37	7	21%	12	32%		
	38–42	11	33%	13	34%		
	43–47	5	15%	6	16%		
	48–52	1	3%	0	0%		
	53 and above	1	3%	2	5%		χ^2^ (4, N = 68) = 6.96
Educational Status	Associate’s degree	2	6%	0	0%	0.16	
	Bachelor’s degree	27	82%	35	92%		
	Graduate degrees	4	12%	3	8%		8
Yoga Experience	I actively practice yoga.	2	6%	1	3%	0.713	
	I know about yoga.	7	21%	9	24%		
	I’ve done yoga before.	1	3%	3	8%		
	I don’t know about yoga/I don’t have yoga practice.	23	70%	25	66%		χ^2^ (1, N = 68) = 13.52

N = Number of participants; df = degrees of freedom; chi^2^ = chi-square test statistic.

**Table 2:** Cognitive Distortion Scale analysis results.

Cognitive Distortion Scale		Intervention Group N = 33 Mean ± SD	Control Group N = 38 Mean ± SD	*p* Value	Group Effect F(df)	ηp^2^	Time Group ΔM (95% CI)	Hedges’ g	ηp^2^
Total Score	Pretest	1.90 ± 0.47	1.78 ± 0.30	0.123					
	Posttest	1.66 ± 0.48	1.79 ± 0.43	*p* < 0.001 *p* < 0.001					
	*p* Value	0.058	0.598		F(1, 68) = 1.11 *p* = 0.296	0.016	ΔM = −0.218 [−0.498, 0.062]	−0.36	0.034
Sub-Dimensions									
s	Pretest	1.83 ± 0.49	1.75 ± 0.58	0.551					
	Posttest	1.83 ± 0.81	1.69 ± 0.53	0.385					
	*p* Value	0.381	0.006		F(1, 68) = 0.28 *p* = 0.599	0.004	ΔM = −0.193 [−0.543, 0.157]	−0.26	0.017
Self-Blame	Pretest	1.72 ± 0.52	1.75 ± 0.58	0.790					
	Posttest	1.79 ± 0.69	1.69 ± 0.53	0.509					
	*p* Value	0.391	0.006		F(1, 68) = 1.48 *p* = 0.227	0.021	ΔM = −0.179 [−0.515, 0.156]	−0.25	0.016
Helplessness	Pretest	1.65 ± 0.44	1.79 ± 0.54	0.252					
	Posttest	1.87 ± 0.69	1.77 ± 0.66	0.561					
	*p* Value	0.046	0.607						
Hopelessness	Pretest	1.59 ± 0.65	1.60 ± 0.61	0.944					
	Posttest	1.38 ± 0.55	1.50 ± 0.55	0.366					
	*p* Value	0.667	0.294		F(1, 68) = 3.16 *p* = 0.080	0.044	ΔM = −0.435 [−0.775, −0.095]	−0.60	0.086
Seeing the Future as Dangerous	Pretest	2.07 ± 0.63	1.88 ± 0.53	0.179					
	Posttest	1.93 ± 0.59	1.89 ± 0.65	0.802					
	*p* Value	0.823	0.238		F(1, 68) = 0.1 *p* = 0.666	0.003	ΔM = 0.017 [−0.393, 0.427]	0.02	*p* < 0.001

**Table 3:** Brief Symptom Inventory analysis results.

Brief Symptom Inventory Subscale		Intervention Group N = 33 Mean ± SD	Control Group N = 38 Mean ± SD	*p* Value	Group Effect F(df)	ηp^2^	Time Group ΔM (95% CI)	Hedges’ g	ηp^2^
Total	Pretest	0.76 ± 0.48	0.47 ± 0.39	*p* < 0.001					
	Posttest	0.62 ± 0.35	0.51 ± 0.41	*p* < 0.001					
	*p* Value	0.027	0.018		F(1, 68) = 0.45 *p* = 0.503	0.007	ΔM = −0.129 [−0.373, 0.115]	−0.25	0.016
Anxiety	Pretest	0.63 ± 0.44	0.40 ± 0.043	0.034					
	Posttest	0.35 ± 0.37	0.40 ± 0.46	0.638					
	*p* Value	0.547	0.727		F(1, 68) = 3.18 *p* = 0.079	0.045	ΔM = −0.006 [−0.260, 0.249]	−0.01	F(1, 68) = 3.18 *p* = 0.079
Depression	Pretest	0.69 ± 0.58	0.53 ± 0.48	0.011					
	Posttest	0.44 ± 0.39	0.49 ± 0.50	0.626					
	*p* Value	0.048	0.806		F(1, 68) = 0.45 *p* = 0.503	0.007	ΔM = −0.220 [−0.536, 0.096]	−0.33	0.027
Negative Self	Pretest	0.69 ± 0.52	0.48± 0.40	0.060					
	Posttest	0.42 ± 0.36	0.41 ± 0.46	0.847					
	*p* Value	0.263	0.820		F(1, 68) = 0.53 *p* = 0.471	0.008	ΔM = −0.095 [−0.349, 0.158]	−0.18	0.008
Somatization	Pretest	0.53 ± 0.46	0.43 ± 0.43	0.386					
	Posttest	0.33 ± 0.34	0.35 ± 0.45	0.888					
	*p* Value	0.016	0.821		F(1, 68) = 1.60 *p* = 0.210	0.023	ΔM = −0.195 [−0.448, 0.058]	−0.36	0.033
Hostilities	Pretest	0.91 ± 0.63	0.61 ± 0.50	0.027					
	Posttest	0.52 ± 0.38	0.52 ± 0.54	0.952					
	*p* Value	0.071	0.926		F(1, 68) = 0.47 *p* = 0.495	0.007	ΔM = −0.171 [−0.475, 0.132]	−0.27	0.018

**Table 4:** Communication Skills Scale analysis results.

Communication Skills Scale		Intervention Group N = 33 Mean ± SD	Control Group N = 38 Mean ± SD	*p* Value	Group Effect F(df)	ηp^2^	Time Group ΔM (95% CI)	Hedges’ g	ηp^2^
Total	Pretest	4.16 ± 0.36	3.91 ± 0.94	*p* < 0.001					
	Posttest	4.22 ± 0.72	3.86 ± 0.96	*p* < 0.001					
	*p* Value	0.161	0.694		F(1, 68) = 4.83 *p* = 0.031	0.066	ΔM = 0.102 [−0.503, 0.708]	0.08	0.002
Subscale									
Communication Principles and Basic Skills at Scale	Pretest	3.60 ± 0.34	3.35 ± 0.90	0.104					
	Posttest	3.34 ± 0.87	3.55 ± 0.65	0.289					
	*p* Value	0.322	0.558		F(1, 68) = 4.57 *p* = 0.036	0.063	ΔM = 0.084 [−0.536, 0.705]	0.06	0.001
Self-Expression	Pretest	3.27 ± 0.36	3.06 ± 0.81	0.209					
	Posttest	3.08 ± 0.78	3.32 ± 0.59	0.131					
	*p* Value	0.912	0.236		F(1, 68) = 0.12 *p* = 0.729	0.002	ΔM = −0.074 [−0.694, 0.546]	−0.06	0.001
Willingness to Communicate and Active Listening	Pretest	4.31 ± 0.46	3.94 ± 1.05	0.069					
	Posttest	3.96 ± 1.00	4.25 ± 0.66	0.136					
	*p* Value	0.195	0.366		F(1, 68) = 7.99 *p* = 0.006	0.105	ΔM = 0.144 [−0.484, 0.771]	0.11	0.003
Non-Verbal Communication	Pretest	3.56 ± 0.38	3.29 ± 0.61	0.088					
	Posttest	3.28 ± 0.83	3.52 ± 0.55	0.187					
	*p* Value	0.235	0.473		F(1, 68) = 6.08 *p* = 0.016	0.082	ΔM = 0.230 [−0.405, 0.865]	0.17	0.008

**Table 5:** Intolerance to Uncertainty Scale analysis results.

Intolerance of Uncertainty Scale		Intervention Group N = 33 Mean ± SD	Control Group N = 38 Mean ± SD	*p* Value	Group Effect F(df)	ηp^2^	Time Group ΔM (95% CI)	Hedges’ g	ηp^2^
Total	Pretest	2.31 ± 0.83	2.16 ± 0.91	0.026					
	Posttest	3.11 ± 0.84	2.26 ± 0.90	0.001					
	*p* Value	0.645	0.153		F(1, 68) = 16.37	0.194	ΔM = 0.724 [0.235, 1.213]	0.69	0.112
Subscale									
Factor 1. Uncertainty is stressful and upsetting.	Pretest	1.83 ± 0.73	2.05 ± 0.90	0.40					
	Posttest	1.83 ± 0.89	2.50 ± 0.92	0.918					
	*p* Value	0.968	0.039		F(1, 68) = 12.35	0.154	ΔM = 0.554 [0.021, 1.088]	0.49	0.059
Factor 2. Negative self-evaluations related to uncertainty.	Pretest	1.72 ± 0.82	1.95 ± 0.80	0.178					
	Posttest	1.79 ± 0.85	2.22 ± 0.87	0.772					
	*p* Value	0.547	0.180		F(1, 68) = 13.23	0.163	ΔM = 0.712 [0.209, 1.215]	0.66	0.104
Factor 3. Not knowing the future is unsettling.	Pretest	1.65 ± 0.86	2.18 ± 0.80	0.111					
	Posttest	1.87 ± 0.96	2.57 ± 1.02	0.998					
	*p* Value	0.885	0.067		F(1, 68) = 14.48	0.176	ΔM = 0.761 [0.269, 1.254]	0.73	0.121
Factor 4. Uncertainty prevents me from taking action.	Pretest	1.59 ± 0.77	1.71 ± 0.61	0.074					
	Posttest	1.38 ± 0.80	2.04 ± 0.74	0.459					
	*p* Value	0.439	0.033		F(1, 68) = 20.39	0.231	ΔM = 0.985 [0.397, 1.573]	0.79	0.139

## Data Availability

The datasets for this study can be accessed at https://osf.io/6eqna/overview?view_only=6f2db9b38f5744dd92fa3154b79e34ed, access date: 1 January 2024). Restrictions apply to the availability of these data. The data were obtained from employees of a public institution and are available with the permission of the institution.
